# DNA binding activities of the *Herves *transposase from the mosquito *Anopheles gambiae*

**DOI:** 10.1186/1759-8753-2-9

**Published:** 2011-06-20

**Authors:** Amandeep S Kahlon, Robert H Hice, David A O'Brochta, Peter W Atkinson

**Affiliations:** 1 Interdepartmental Graduate Program in Cell, Molecular and Developmental Biology, University of California, Riverside, CA, USA; 2 Institute of Integrative Genome Biology and Department of Entomology, University of California, Riverside, CA, USA; 3 Department of Entomology and Institute for Bioscience and Biotechnology Research, University of Maryland, Rockville, MD, USA

## Abstract

**Background:**

Determining the mechanisms by which transposable elements move within a genome increases our understanding of how they can shape genome evolution. Class 2 transposable elements transpose via a 'cut-and-paste' mechanism mediated by a transposase that binds to sites at or near the ends of the transposon. *Herves *is a member of the *hAT *superfamily of class 2 transposons and was isolated from *Anopheles gambiae*, a medically important mosquito species that is the major vector of malaria in sub-Saharan Africa. *Herves *is transpositionally active and intact copies of it are found in field populations of *A gambiae*. In this study we report the binding activities of the *Herves *transposase to the sequences at the ends of the *Herves *transposon and compare these to other sequences recognized by *hAT *transposases isolated from other organisms.

**Results:**

We identified the specific DNA-binding sites of the *Herves *transposase. Active *Herves *transposase was purified using an *Escherichia coli *expression system and bound in a site-specific manner to the subterminal and terminal sequences of the left and right ends of the element, respectively, and also interacted with the right but not the left terminal inverted repeat. We identified a common subterminal DNA-binding motif (CG/AATTCAT) that is critical and sufficient for *Herves *transposase binding.

**Conclusions:**

The *Herves *transposase binds specifically to a short motif located at both ends of the transposon but shows differential binding with respect to the left and right terminal inverted repeats. Despite similarities in the overall structures of *hAT *transposases, the regions to which they bind in their respective transposons differ in sequence ensuring the specificity of these enzymes to their respective transposon. The asymmetry with which the *Herves *terminal inverted repeats are bound by the transposase may indicate that these differ in their interactions with the enzyme.

## Background

Transposable elements (TEs) are ubiquitous components of genomes in which they impact genomic evolution and maintenance [1-6]. Their mobility properties have resulted in their adoption as genetic tools in modern genetics with one of their many uses in biotechnology being the introduction of foreign genes into insect disease vectors of medical and agricultural importance [7-14]. *Anopheles gambiae *is the principal vector of the malaria-causing parasite *Plasmodium falciparum *in sub-equatorial Africa and is a mosquito species in which robust TE-based genetic tools need to be developed. At present there are six reports of successful genetic transformation of this mosquito, one using the *P *element, and five using the *piggyBac *element, transformation remaining a low frequency event [9,15-19]. Isolating active, well adapted, endogenous TEs from *A gambiae *and understanding their biology is likely to improve the efficiency of genetic transformation in this species since these native active TEs are likely to have adapted to overcome or evade the host response systems that are proposed inactivate mobile DNA [20,21].

**Figure 1:**
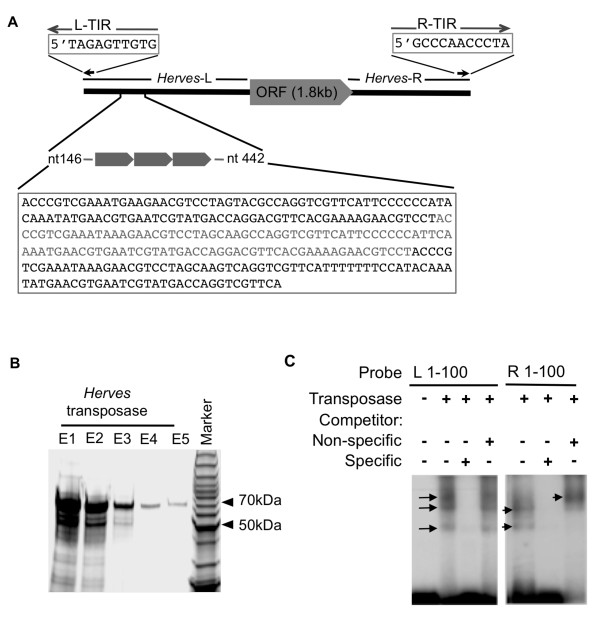
***Herves *transposase binds to the terminal sequences of L and R ends of the *Herves *element**. **(a) **Schematic representation of the *Herves *element. Numbers indicate the distance in bp internal to either the left (L) or right (R) end. **(b) **SDS-PAGE analysis of purified *Herves *transposase. A Coomassie-stained gel shows 70 kDa purified *Herves *transposase. E1-E5 represent different elutions obtained during the final step of protein purification. **(c) **Transposase binding to the terminal fragment of *Herves*-L and *Herves*-R ends. Electrophoretic mobility shift assay (EMSA) analysis with the *Herves*-L bp 1-100 (lanes 1-4) and *Herves*-R bp 1-100 (lanes 5-7) probes. The DNA fragments were incubated in the presence (+) or absence (-) of pure transposase. A homologous fragment was used as specific competitor. The E1 flanking sequence from the *Hermes *transposable element was used as the non-specific competitor [44]. Specific and non-specific competitors were use at 200-fold molar excess to the probe. Arrows indicate various protein DNA complexes.

*Herves *is an active class 2 TE that was isolated from *A gambiae *[22]. It contains a transposase-encoding open reading frame (ORF) that is flanked by left (*Herves*-L) and right (*Herves*-R) end sequences with the *Herves*-L end being unusually long (1,478 bp) compared with the *Herves*-R end (421 bp) and contains three 100 bp imperfect tandem repeats, commencing 146 bp from the end (Figure 1a). *Herves *has 11 bp imperfect terminal inverted repeats (TIRs) at the left (L-TIR) and right (R-TIR) ends (Figure 1a) [22]. It is transpositionally active and can genetically transform *Drosophila melanogaster *[22]. Population dynamics studies suggest that *Herves *has been recently active within field populations of *A gambiae *from Kenya and that many intact copies of it are present in these populations [23]. Class 2 TEs often accumulate internal deletions over time that render the elements inactive and so unable to cause further harm to the host organism [24]. The presence of intact forms of *Herves *and other *hAT *TEs, such as *Hermes*, indicate that at least some *hAT *elements appear, for reasons unknown, less prone to accumulating internal deletions however the significance of this in absence of information concerning MITEs (Miniature Inverted Terminal Elements) generated from them remains unknown [23,25,26].

Class 2 transposases typically bind to the TIRs and nearby internal sequences and mediate transposition to a new genomic location by the classical 'cut-and-paste' mechanism [27]. Other *cis*-acting sequences, which usually consist of short repeat sequence motifs located close to the TIRs, are also important for proper transposase binding and efficient excision and transposition [28-35]. In many cases, native *cis *elements are not optimized for maximal transposition mobility; thus, new and improved TE gene vectors can be designed by altering these elements to increase or decrease transposase binding [36,37]. The identification and characterization of these transposase binding sites and of the specific DNA-binding transposase residues is therefore important to our understanding of the biology and post integration behavior of TEs. This study aimed to identify the DNA sequences of the *Herves *element bound by its transposase.

## Results

### Purification of *Herves *transposase and its binding to the *Herves*-L end

*Herves *transposase is 603 amino acids in length and is predicted to have a molecular weight of 70 kDa. *Herves *transposase was purified from an *Escherichia coli *expression system and its size was confirmed by SDS-PAGE (Figure 1b). To examine the binding of *Herves *transposase to the *Herves*-L end, we focused on the terminal 100 bp region. A radioactively labeled *Herves*-L 1-100 bp probe was incubated in the presence or absence of purified *Herves *transposase for use with a molar excess (200-fold) of unlabeled specific and non-specific DNA fragments were used as specific and non-specific competitors, respectively, in electrophoretic mobility shift assays (EMSAs). The transposase interacted with the *Herves*-L 100 bp probe and formed three transposase-DNA complexes (Figure 1c). A specific competitor competed for the transposase, but the non-specific competitor did not affect binding (Figure 1c) implicating a sequence-specific interaction between the transposase and the probe.

**Figure 2:**
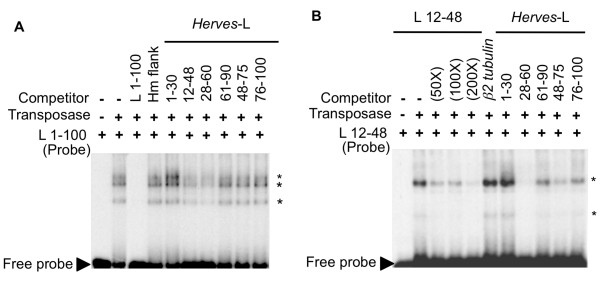
***Herves*-L bp 12-48 and bp 28-60 are important for transposase binding**. Electrophoretic mobility shift assays (EMSAs) with **(a) ***Herves*- left (L) bp 1-100 and **(b) ***Herves*-L bp 12-48 as probes. Overlapping 30 bp fragments were used as competitors for transposase binding to the probe. The specific and non-specific competitors were use at 200-fold molar excess to the probe, unless specified otherwise. The asterisk (*) indicates various protein DNA complexes. **(a) **Specific and non-specific competitors was used as described for Figure 1; **(b) **a 30 bp non-homologous fragment, from the *β2 tubulin *gene of *Aedes aegypti*, was used as a non-specific competitor.

To specify the transposase binding site(s) within this terminal 100 bp sequence, overlapping oligonucleotides (approximately 30 bp in length) were competed with the *Herves*-L 100 bp probe for transposase binding. The DNA fragments *Herves*-L bp 12-48 and bp 28-60 competed with this probe in all three transposase-DNA complexes, whereas the *Herves*-L bp 1-30, bp 48-75, and bp 76-100 fragments had no effect (Figure 2a). This suggested that the *Herves *transposase binds tightly and specifically within the L bp 12-60 region. The overlapping *Herves*-L bp 1-30 and bp 48-75 fragments did not alter binding, indicating that a binding motif(s) was present in the *Herves*-L bp 28-48 bp (Figure 2a). We also observed that the L bp 12-48 bp and bp 28-60 fragments competed partially with the 100 bp probe, whereas the specific bp 1-100 fragment competed fully for transposase binding, implicating the existence of additional binding motifs that act cooperatively with the binding motif(s) in the bp 28-48 region (Figure 2a).

To confirm these results, each of the unlabeled 30 bp fragments was tested against the *Herves*-L bp 12-48 probe for binding to the transposase. Binding to the transposase was observed for this probe (Figure 2b), which resulted in two transposase-DNA complexes. The unlabeled L bp 28-60 fragment specifically competed for binding of the transposase (Figure 2b). *Herves*-L bp 48-75, bp 61-90, and bp 76-100 competed partially with the probe, indicating weak transposase binding to these regions. These results suggested that *Herves*-L bp 12-48 and bp 28-60 have strong and equal binding for *Herves *transposase, leading us to believe that the DNA binding motif lay within the *Herves*-L bp 28-48 fragment.

**Figure 3:**
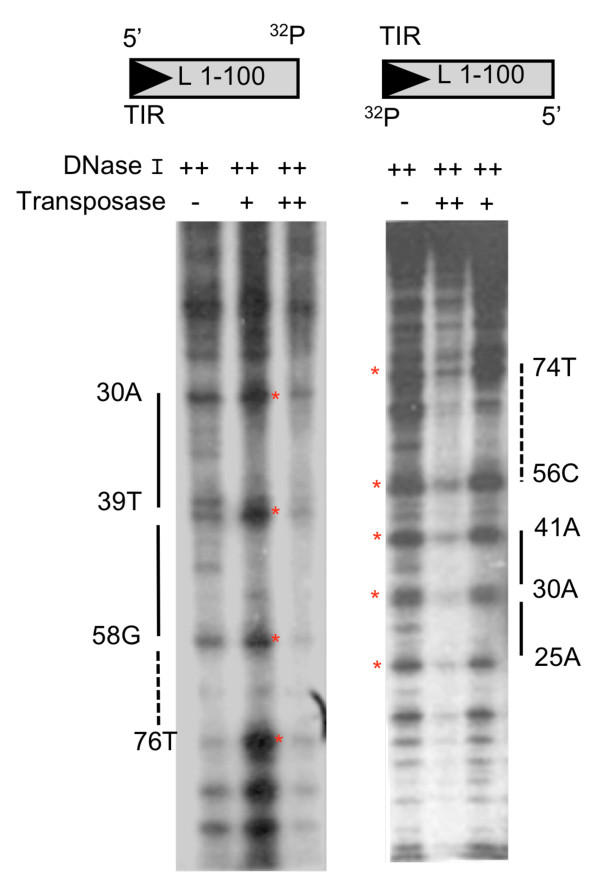
**Transposase binding analysis to the *Herves*-left (L) end**. The single-end-labeled *Herves*-L 1-100 bp fragment (100 nM) was incubated in presence (+, ++) or absence (-) of DNase I or the transposase. The ++ indicates 1.4 μM of transposase or 0.2 units of DNase I, whereas + indicates 850 nM of transposase or 0.1 units of DNase I. ^32^P indicates the position where probe was labeled. The solid bars indicate regions protected by the transposase from DNase I degradation. The red asterisk (*) indicates hypersensitive sites.

We performed DNase I protection assays to confirm the EMSA results and to specifically identify the DNA region bound by pure transposase. A terminal 1-100 bp fragment was labeled at the 3' end and labeled probes were incubated separately with *Herves *transposase and subsequently with DNase I and then analyzed on a denaturing polyacrylamide gel. The two 3' end-labeled probes were protected at bp 25-73 and bp 30-75, respectively (Figure 3). Increasing amounts of transposase led to greater protection of the *Herves*-L 100 bp probe (Figure 3). Overall, the DNase I protection assay results confirmed the EMSA findings, indicating sequence-specific binding of transposase to at least the *Herves*-L bp 28-48 region.

### Transposase binds to the *Herves*-R end

To investigate the binding of transposase to the *Herves*-R end, the *Herves*-R 1-100 bp fragment was radiolabeled and used in EMSAs. *Herves *transposase interacted specifically with the probe and formed two transposase-DNA complexes (Figure 1c). Unlabeled specific competitor competed with the probe for transposase and, notably, the addition of a non-specific competitor led to the formation of a single, higher-molecular-weight complex (Figure 1c). The molecular composition of this complex, however, is unknown.

**Figure 4:**
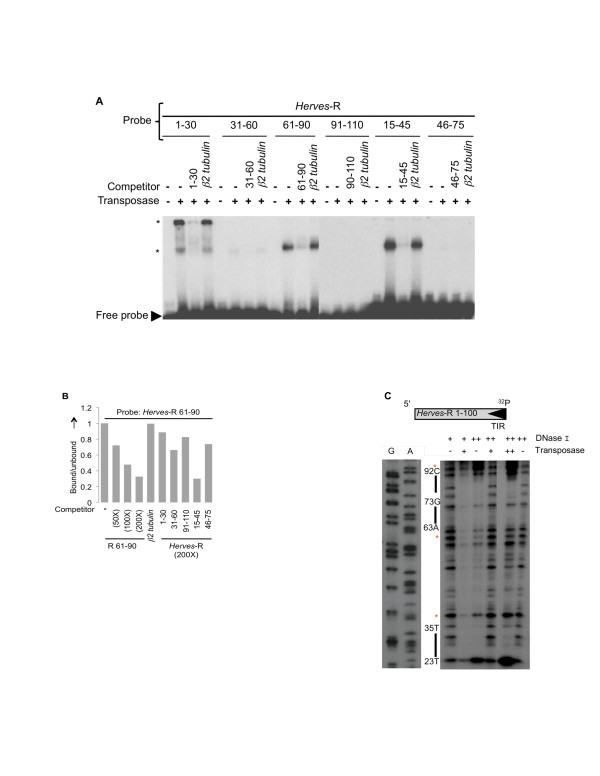
**Transposase binding to the *Herves*-right (R) end**. **(a) ***Herves *transposase binds to *Herves*-R bp 1-30, bp 5-45 and bp 61-90. Electrophoretic mobility shift assay (EMSA) analysis of transposase binding to the overlapping 30 bp fragments (bp 1-30, bp 31-60, bp 61-90, bp 91-110, bp 15-45 and bp 46-75). The asterisk (*) indicates various protein DNA complexes. **(b) ***Herves *transposase binding to *Herves*-R bp 15-45 and bp 61-90. The *Herves*-R bp 61-90 fragment was used as a probe in EMSAs. The fraction of the transposase-bound probe was quantified using a phosphoimager. A homologous fragment was used as specific competitor at a molar excess of 50-fold, 100-fold and 200-fold, whereas non-specific competition was used as described for Figure 2b. Overlapping fragments (bp 1-30, bp 31-60, bp 91-110, bp 15-45, and bp 46-75) were used as competitors of transposase binding to the probe, at 200-fold molar excess. **(c) **DNase I protection assay of the *Herves*-R end. The single-end-labeled *Herves*-R 1-100 bp fragment (100 nM) was incubated in presence (+, ++) or absence (-) of DNase I or the transposase. The ++ indicates 1.4 μM of transposase or 0.5 units of DNase I, whereas + indicates 850 nM of transposase or 0.25 units of DNase I. ^32^P indicates the end of the probe that was labeled. The solid bars on the sides indicate the region of the probe protected by the transposase. The asterisk (*) indicates hypersensitive sites.

Overlapping 30 bp oligonucleotides were then used as probes to identify the transposase binding site(s) within the *Herves*-R 1-100 bp region by EMSA. The bp 1-30, bp 15-45, and bp 61-90 fragments elicited specific binding of transposase (Figure 4a). Fragment bp 31-60 showed weak, non-specific binding, whereas the bp 46-75 and bp 91-110 fragments failed to bind (Figure 4a).

Two transposase-DNA complexes formed with the *Herves*-R bp 1-30, compared with a single complex each with the *Herves*-R bp 15-45 and bp 61-90 fragments, implicating the existence of two transposase binding sites within *Herves*-R bp 1-30 fragment and one site within both the *Herves*-R bp 15-45 and bp 61-90 fragments (Figure 4a). To determine relative transposase binding preferences, each 30 bp overlapping DNA fragment was allowed to compete against the *Herves*-R bp 61-90 probe for transposase binding using EMSAs. Fragment bp 15-45 successfully competed against the probe for transposase, whereas the *Herves*-R bp 1-30 and bp 31-60 fragments had no effect (Figure 4b). These data suggest that the transposase binds strongly to the terminal *Herves*-R end at positions bp 15-45 and bp 61-90.

We performed DNase I protection assays to identify specific binding motifs in the R end of *Herves *however these were inconclusive and showed some evidence of protection at bp 23-35 and bp 63-92 (Figure 4c).

### Mutational analysis of the *Herves *transposase binding motif

**Figure 5:**
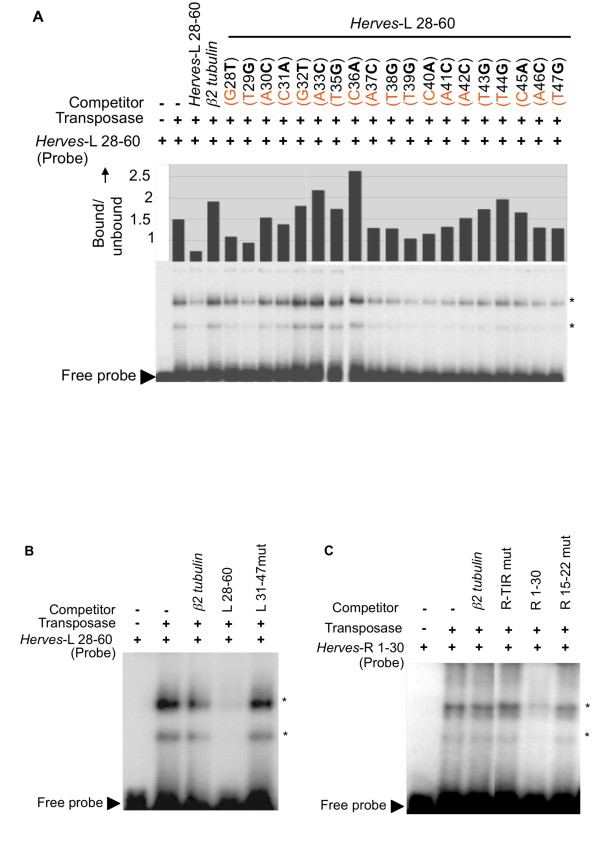
**CGATTCAT acts as transposase binding motif**. **(a) **Electrophoretic mobility shift assay (EMSA) analysis of transposase binding to the *Herves*-left (L) bp 28-60 probe and single nucleotide sequence variants as competitors. For example: G28**T **indicates that G at position 28 was changed to T in the *Herves*-L bp 28-60 fragment. The fraction of the transposase bound probe in each lane was quantified using a phosphoimager. Mutations that have no effect on the transposase binding are expected to produce values similar to the specific competitor. **(b) **The *Herves*-L 31-47 region is important for binding. The asterisk (*) indicates various protein DNA complexes. The *Herves*-L 28-60 bp fragment was used as a probe in EMSA experiment. The *Herves*-L 31-47mut carries mutations at every position within bp 31-47. **(c) **Role of right (R)-terminal inverted repeat (TIR) in the transposase binding. The *Herves*-R bp 1-30 fragment was used as a probe. The *Herves*-R TIRmut and R 15-22mut fragments consist of the *Herves*-R bp 1-30 sequence with mutations in TIR and bp 15-22 respectively. The probe was incubated in the presence (+) or absence (-) of the transposase or competitors. Unlabeled homologous and non-homologous fragments were used as specific and non-specific competitors, respectively. The asterisk (*) indicates various protein DNA complexes.

Because the *Herves*-L bp 28-48 fragment showed the strongest binding to transposase, a detailed analysis was performed to define the critical nucleotides for binding. We analyzed 22 sequence variants for their ability to compete with the *Herves*-L bp 28-60 probe for transposase. Each sequence variant differed from the wild-type sequence by a single nucleotide. An unlabeled wild-type *Herves*-L bp 28-60 fragment competed successfully against the probe for transposase binding, whereas mutating nucleotides *Herves*-L bp 32-36 and bp 43-45 abolished this competition, indicating that nucleotides at these positions mediate the binding of transposase (Figure 5a). We identified a conserved binding motif, CG/AATTCAT, in both regions, suggesting that it constitutes the transposase-binding motif. To confirm these results, we simultaneously mutated this putative motif at both locations within the *Herves*-L bp 28-60 fragment and allowed the mutant (*Herves*-L 31-47mut) to compete against the wild-type *Herves*-L bp 28-60 probe. Mutating both sites abolished the interaction, confirming that CG/AATTCAT is the binding site for *Herves *transposase in the *Herves*-L end (Figure 5b).

### The CG/AATTCAT motif is conserved between the *Herves*-L and *Herves*-R ends

We identified similar potential binding motifs within the *Herves*-R bp 15-22 and bp 73-86 regions. Furthermore, the bp 1-30 region also contains the R-TIR, a potential candidate for transposase binding. To determine whether the R-TIR or the CG/AATTCAT motif mediated the binding of transposase to the *Herves*-R bp 1-30 region, we mutated each region (*Herves*-R TIRmut and *Herves*-R bp 15-22mut) and subjected them to EMSA. Mutating each potential binding site abolished its ability to compete against the wild-type probe, suggesting that the CG/AATTCAT motif and R-TIR are both important for the transposase binding to the *Herves*-R end (Figure 5c).

### The CGATTCAT motif is sufficient for purified *Herves *transposase binding

**Figure 6:**
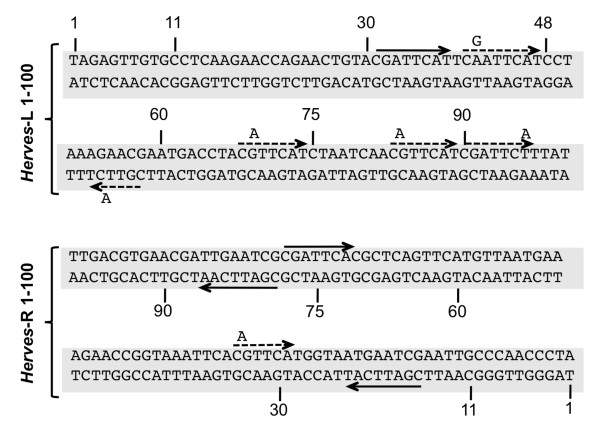
**The CGATTCAT binding motif is sufficient for transposase binding**. The probe (CGATTCAT)_4 _represents four direct repeats of the CGATTCAT sequence motif. Unlabeled (CGATTCAT)_4 _and *Herves*-left (L) bp 28-60 were used as specific competitors, whereas *β2 tubulin *was used as the non-specific competitor. The transposase binding was compared between the sequence variants of the binding motif such as CGATTCTT, CGATTCAC and CGTTCAT (each used as four direct repeats). The asterisk (*) indicates various protein DNA complexes.

**Figure 7:**
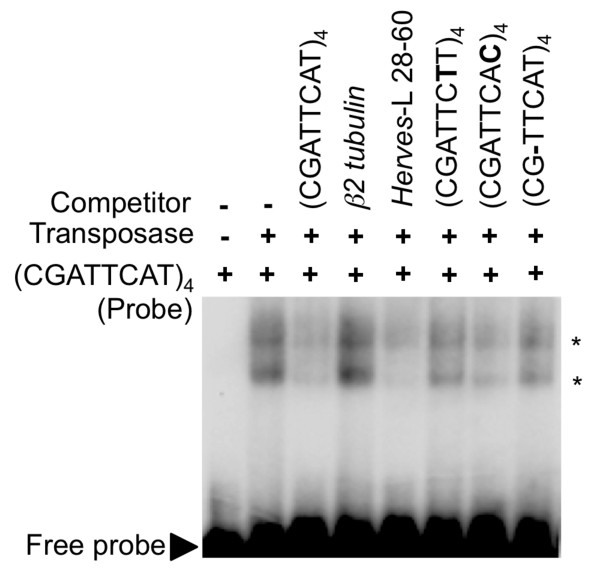
**Sequence of *Herves*-left (L) bp 1-100 and *Herves*-right® bp 1-100 showing sequence repeats**. The solid arrow indicates conserved CGATTCA transposase binding motif, whereas the dotted arrow indicates the single nucleotide sequence variants.

The CG/AATTCAT motif and its derivatives are repeated several times within the transposase binding regions at both the *Herves*-L and *Herves*-R ends (Figure 6). To determine whether the CG/AATTCAT motif was sufficient for transposase binding, we used a probe containing four direct repeats of the CGATTCAT sequence as a probe to measure relative binding to the *Herves *transposase. The transposase bound to the (CGATTCAT)_4 _probe and formed two transposase-DNA complexes (Figure 7). Based on the unlabeled specific and non-specific competitors, the interaction was determined to be sequence specific.

We used *Herves*-L bp 28-60 as a specific competitor for transposase against the (CGATTCAT)_4 _probe and found that it outcompeted it for transposase (Figure 7). Furthermore, splitting the CGATTCAT motif in half abolished the binding (data not shown). Together, these data indicated that the CGATTCAT motif was sufficient for the transposase binding.

We also tested the ability of unlabeled sequence variants of the CGATTCAT motif (CGATTC**T**T/CGATTCA**C**/CGTTCAT) to compete against radiolabeled CGATTCAT for transposase binding. None of the sequence variants competed fully with CGATTCAT for the transposase, indicating that CGATTCAT is the strongest binding motif (Figure 7). Nevertheless, CGATTCAC competed partially for transposase, suggesting that this variant may also be important for binding of transposase.

## Discussion

We purified active *Herves *transposase and demonstrated that it site-specifically binds to subterminal and terminal sequences at the *Herves*-L and *Herves*-R ends, respectively. Such asymmetrical binding may affect transposition frequency. The *Drosophila P *element transposase has been shown to bind asymmetrically to the *P *ends and interchanging the L end sequence with the R end sequence led to fewer transposition events [38]. This phenomenon also occurs for the *Ac *element in maize and the *Tag1 *element in *Arabidopsis *[28,31].

There was strong transposase binding to the *Herves*-L bp 12-48 and bp 28-60 regions and relatively weak binding to bp 48-75 as shown by EMSA and DNase I footprinting. None of these fragments however, outcompeted the L bp 1-100 probe for transposase binding, suggesting that the binding was cooperative between two or more regions. Furthermore, the overlapping *Herves*-L bp 12-48 and bp 28-60 fragments showed similar levels of binding, indicating that the binding motif lies in the overlapping region in *Herves*-L bp 28-48. In contrast to the L end, the binding occurred toward the terminal sequences on the *Herves*-R end in regions bp 15-45 and bp 61-90.

EMSA results with the *Herves*-L bp 28-60 probe and single nucleotide sequence variants indicated that the CGATTCAT motif, or its derivatives, mediated binding of the transposase. The CGATTCAT transposase-binding motif and its derivatives are repeated and conserved in the *Herves*-L and *Herves*-R end sequences. Our results suggested that this motif is important and sufficient for transposase binding, because: (1) mutating the CGATTCAT motif at either end abolished binding, and (2) the transposase bound specifically to a synthetic tetramer of the motif.

TEs frequently have multiple transposase binding sites adjacent to their TIRs [33,39-42]. In other *hAT *elements, such as *Ac, Tol2 *and *Tag1*, their respective transposases bind to short sequence repeats [31,33,34,43]. For *Ac *and *Tol2*, the transposase binding sequence motifs differ at the L and R ends [33,34]. The *Herves *transposase-binding CGATTCAT motif, however, is highly conserved at both ends with several single nucleotide variants CGATTCAC, CGTTCAT, and CGATTCTT being present. Our results suggest that these additional motifs may also mediate transposase binding. Although these derivatives are related to the CGATTCAT motif, their ability to bind transposase differs. The transposase binds to CGATTCAT, but weakly to the CGATTCAC and CGTTCAT motifs. It is also possible that the transposase only recognizes a subset or a family of related sequences in which GATTC or ATTCA is the central sequence. Similar results have been reported for the *Tag1 *element, for which the R-TGACCC and L-AAACCC motifs have different affinities for the transposase [31,43]. The sequences that flank these motifs differ, and although they might fail to influence transposase binding, they may regulate transposition [31].

We observed no binding to the L-TIR. Several related *hAT *transposases, such as *Ac *and *Tag1*, do not bind their L-TIR and R-TIR sequences [33,43]. This phenomenon raises the possibility that transposase binding to the L-TIR may require the presence of a host factor however nuclear extracts from a *Herves *transposase-expressing *Drosophila *S2 cell line did not bind to L-TIR making the argument for such a factor less compelling. Nevertheless, pure *Herves *transposase interacted with the R-TIR sequence, the binding at which appeared to be cooperative since both the R-TIR and CGATTCAT motif at 15-22 bp participated in it.

We have identified the sites within the *Herves *element to which the *Herves *transposase binds and shown that it binds asymmetrically to sequences at either end of the element. Future work will be directed towards determining whether mutants of *Herves *which show changes in the binding of the transposase will affect the transpositional activity of *Herves in vivo *leading to the development of this endogenous TE of *A gambiae *as a genetic tool in this medically important mosquito species.

## Conclusions

We identified the specific DNA-binding sites of the *Herves *transposase, a member of the *hAT *transposon superfamily. We found that it displayed an asymmetry of specific binding to the L and R ends of the *Herves *transposon in that it bound to both subterminal regions but interacted only with the R, but not L, TIR. We identified a common subterminal DNA-binding motif (CG/AATTCAT) that is critical and sufficient for *Herves *transposase binding. The asymmetry of binding of the transposase to the L and R ends may indicate that these ends differ in their interactions with the enzyme during the transposition reaction. The differences in transposase binding sites between different *hAT *transposases illustrates that this superfamily provides a fascinating diversity with which to study the biology of transposition.

## Methods

### Plasmid constructions

The *Herves *ORF was cloned into pBAD myc/HisA (Invitrogen, Carlsbad, CA). The *Bsp*HI (incorporated into the *Herves *start codon) and *Kpn*I restriction sites were used to amplify a 766-bp fragment of the *Herves *ORF using the *Herves*F-*Bsp*HI (GATCAATCATGATGGCTCCAACAAACGCAAC) and *Herves*R-*Kpn*I (GTTCAAGGTACCTTGAATCCAATTAGCTATATTCTTACC) primers.

The resulting fragment was cloned into *Nco*I*/Kpn*I-digested pBAD myc/HisA to generate pBADHvPCR1. The remaining *Herves *ORF (1,118 bp) was amplified using the *Herves*F-*Kpn*I (CAAGGTACCTTGAACAAATTTGACATAGAGGATAAG) and *Herves*R-*Hin*dIII primers (TATCAAGCTTTGAACAAATTTGACATAGAGGATAAG) and cloned into *Kpn*I/*Hin*dIII digested pBADHvPCR1 to generate pBADHv1.

### *Herves *transposase purification

*Herves *transposase was purified by *His*-tag purification as described [47]. pBADHv1-transformed LMG 194 *E coli *cells were grown overnight at 30°C in LB media that contained carbenicillin (100 mg/ml). The overnight culture was diluted 1:100 in LB and carbenicillin (100 mg/ml) and grown at 30°C and 230 rpm to an absorbance of 0.6 at 600 nm. The cultures were then induced with 0.1% L-arabinose and shaken at 16°C for 18 h. The cells were harvested and washed by centrifugation with binding buffer (0.5 M NaCl, 20 mM Tris-Cl pH 7.9, 10% glycerol, 10 mM imidazole). The cells were lysed twice using a French press at 20,000 psi. The cell lysate was cleared by centrifugation and by passing through 0.45 μm syringe filters. Cleared lysate was loaded onto Sepharose (Amersham/GE Healthcare, Piscataway, NJ) chromatography columns that were pre-equilibrated with Ni^2+^. The columns were washed with 10 ml binding buffer and 6 ml wash buffer (0.5 M NaCl, 20 mM Tris-Cl pH 7.9, 10% glycerol, 50 mM imidazole). His-tagged *Herves *was eluted in five 1 ml fractions of elution buffer (0.5 M NaCl, 20 mM Tris-Cl pH 7.9, 10% glycerol, 200 mM imidazole). The purified *Herves *transposase was dialyzed overnight in dialysis buffers 1 (0.5 M NaCl, 20 mM Tris base, 10% glycerol pH 8.0) and 2 (0.5 M NaCl, 20 mM Tris base, 2 mM dithiothreitol (DTT), 25% glycerol pH 8.0) for 3 h using a Slide-A-Lyzer dialysis cassette (Thermo Fisher Scientific, Waltham, MA). The dialyzed, purified *Herves *transposase was stored at -80°C.

### EMSAs

The DNA fragment (100 nM) that we tested for transposase binding was end labeled using T4 polynucleotide kinase and ^32^P ATP and purified on a Biospin 30 column (BioRad, Hercules, CA). The labeled DNA fragment (probe) was incubated at 4°C for 45 min with 1 × EMSA binding buffer (16 mM Tris pH 8.0, 0.2 μg bovine serum albumin (BSA), 0.4 μg T3 single-stranded oligo, 0.5 μg poly(dI-dC), 1 mM DTT, 150 mM NaCl, 0.25% Triton X) and 850 nM of *Herves *transposase. Specific and non-specific DNA fragments were used as specific and non-specific competitors, respectively (if applicable). The reaction was incubated with the probe for an additional 40 min at 4°C. The non-specific competitors were 126 bp gDNA fragment (E1) that flanks *Hermes *TE from *Musca domestica *and a 30 bp DNA oligo from *Aedes aegypti *β2 tubulin. The EMSA reaction products were analyzed on a 5% TBE polyacrylamide gel (Bio-Rad).

### DNase I protection assay

DNA fragments (100 bp each) from the *Herves*-L and *Herves*-R ends, containing an *Eco*RV restriction site at the L-end or R-end, were cloned into pJET 1.2 (Fermentas/Thermo Fisher Scientific, Piscataway, NJ) to generate pL5'*Eco*RV, pL3'*Eco*RV, pR5'*Eco*RV, and pR3'*Eco*RV. The transferred and non-transferred strands from the *Herves*-L and *Herves*-R ends were selectively radiolabeled at one end by digesting pL5'*Eco*RV, pL3'*Eco*RV, pR5'*Eco*RV, and pR3'*Eco*RV with *Xho*I and *Eco*RV and labeling them with [^32^P] dATP using Klenow (NEB, Ipswich, MA). *Herves *transposase was allowed to bind to 100 nM single end-labeled DNA fragment (probe) under the same binding conditions as in the EMSA. The optimal concentrations of transposase were determined empirically (Additional files 1 and 2). The DNA probe was subjected to DNase I digestion for 2 min at 4°C. The reaction was stopped by adding stop solution (92% ethanol, 0.7 M ammonium acetate, 0.35 μg tRNA) for 15 min in a dry ice/ethanol bath. DNA was extracted with phenol/chloroform and precipitated with ethanol. The reaction products were analyzed on a 10% denaturing polyacrylamide sequencing gel. The DNA sequencing kit 2.0 (USB) was used to construct a nucleotide ladder that was analyzed with the reaction products on the sequencing gel (Additional file 3).

## Competing interests

The authors declare that they have no competing interests.

## Authors' contributions

ASK performed the experiments and wrote early drafts of the manuscript. RHH provided technical expertise and assisted in experimental design. DAO participated in experimental design and edited the manuscript. PWA conceived the study, directed the experimentation and edited subsequent drafts of the manuscript. All authors read and approved the final manuscript.

## Supplementary Material

Additional file 1 **DNase I protection of the *Herves *right (R) end**. Various concentrations of *Herves *transposase (as indicated) were tried to titrate for the optimum concentration for the protections assays for the *Herves *right end. Concentrations higher than 850 nM (such as 1 μM or 1.2 μM) or lower than 850 nM (150 nM, 300 nM and 428 nM) produced non-specific protection of the probe or no protection at all, respectively. **(a) **100 nM or **(b) **50 nM and 100 nM of the single-end-labeled *Herves*-R 1-100 bp fragment was incubated in absence (-) or presence of the transposase at various concentrations as indicated. ^32^P indicates end of the probe that was labeled. Click here for file

Additional file 2 **DNase protection of the *Herves *left (L) end**. Various concentrations of *Herves *transposase (as indicated) were tried to titrate for the optimum concentration for the protections assays for the *Herves *left end. Concentrations higher than 850 nM (such as 1 μM or 1.2 μM) or lower than 850 nM (150 nM, 300 nM and 428 nM) produced non-specific protection of the probe or no protection at all, respectively. **(a) **50 nM or **(b) **100 nM of the single-end-labeled *Herves*-L 1-100 bp fragment was incubated in absence (-) or presence of the transposase at various concentrations as indicated. ^32^P indicates end of the probe that was labeled. Click here for file

Additional file 3 **Figure **3**with DNA ladder**. The panel is identical to that shown in the left of Figure 3 but with a DNA ladder. The nucleotide positions were determined by the Sanger sequencing reactions shown in lanes G and A. Click here for file
