# Supplementary material for: DNA binding activities of the Herves transposase from the mosquito Anopheles gambiae
Source: Mob DNA. 2011 Jun 20;2:9. doi: 10.1186/1759-8753-2-9 (PMC3143072; doi:10.1186/1759-8753-2-9)
Supplement: Additional file 1 — DNase I protection of the Herves right (R) end. Various concentrations of Herves transposase (as indicated) were tried to titrate for the optimum concentration for the protections assays for the Herves right end. Concentrations higher than 850 nM (such as 1 μM or 1.2 μM) or lower than 850 nM (150 nM, 300 nM and 428 nM) produced non-specific protection of the probe or no protection at all, respectively. (a) 100 nM or (b) 50 nM and 100 nM of the single-end-labeled Herves-R 1-100 bp fragment was incubated in absence (-) or presence of the transposase at various concentrations as indicated. 32P indicates end of the probe that was labeled. [file 1759-8753-2-9-S1.PDF]

Additional File 1: Figures S1A and S1B

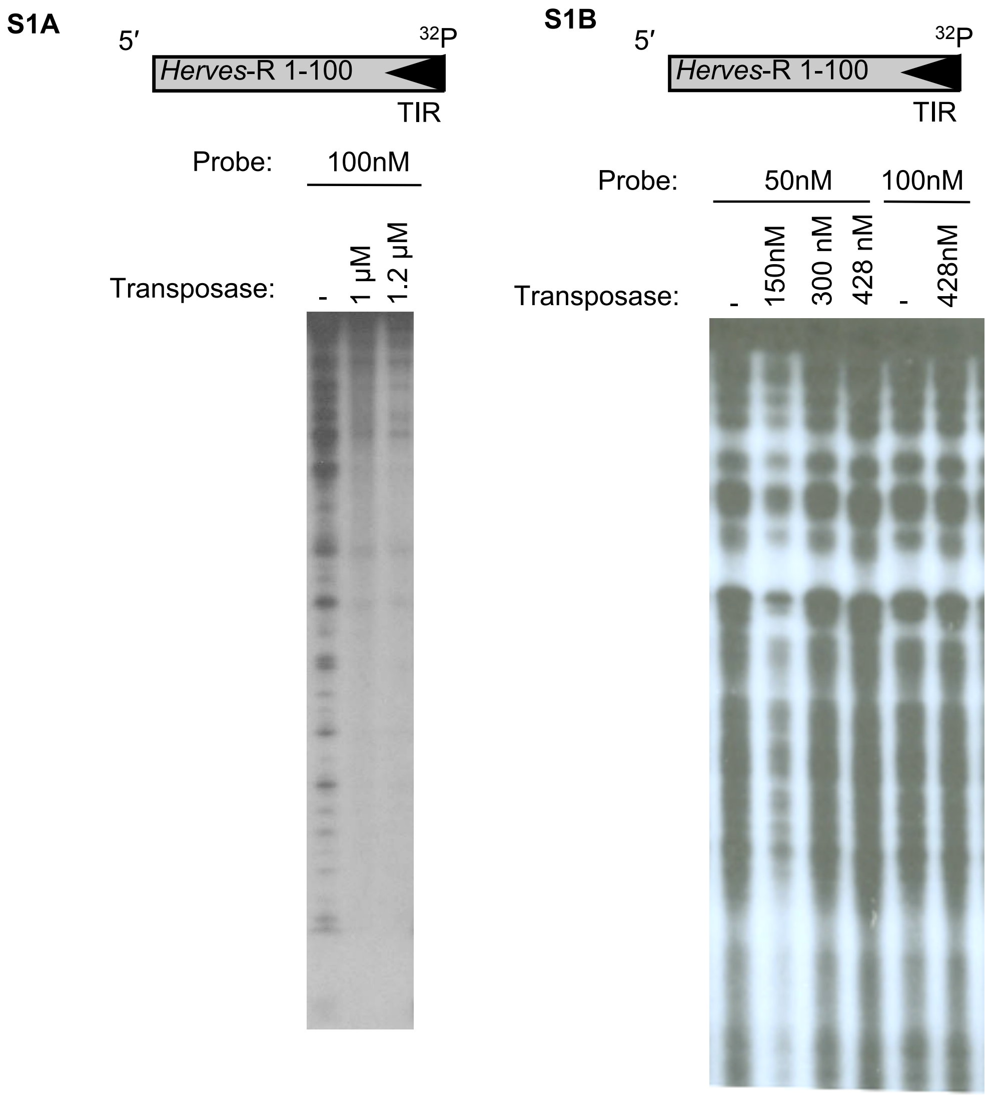

Figure S1.

A) 100nM or **B)** 50nM and 100nM of the single-end labeled *Herves*-R 1-100bp fragment was incubated in absence (-) or presence of the transposase at various concentrations as indicated. ^{32}P indicates end of the probe that was labeled.
