# Supplementary material for: DNA binding activities of the Herves transposase from the mosquito Anopheles gambiae
Source: Mob DNA. 2011 Jun 20;2:9. doi: 10.1186/1759-8753-2-9 (PMC3143072; doi:10.1186/1759-8753-2-9)
Supplement: Additional file 2 — DNase protection of the Herves left (L) end. Various concentrations of Herves transposase (as indicated) were tried to titrate for the optimum concentration for the protections assays for the Herves left end. Concentrations higher than 850 nM (such as 1 μM or 1.2 μM) or lower than 850 nM (150 nM, 300 nM and 428 nM) produced non-specific protection of the probe or no protection at all, respectively. (a) 50 nM or (b) 100 nM of the single-end-labeled Herves-L 1-100 bp fragment was incubated in absence (-) or presence of the transposase at various concentrations as indicated. 32P indicates end of the probe that was labeled. [file 1759-8753-2-9-S2.PDF]

Additional File 2, Figures S2A and S2B

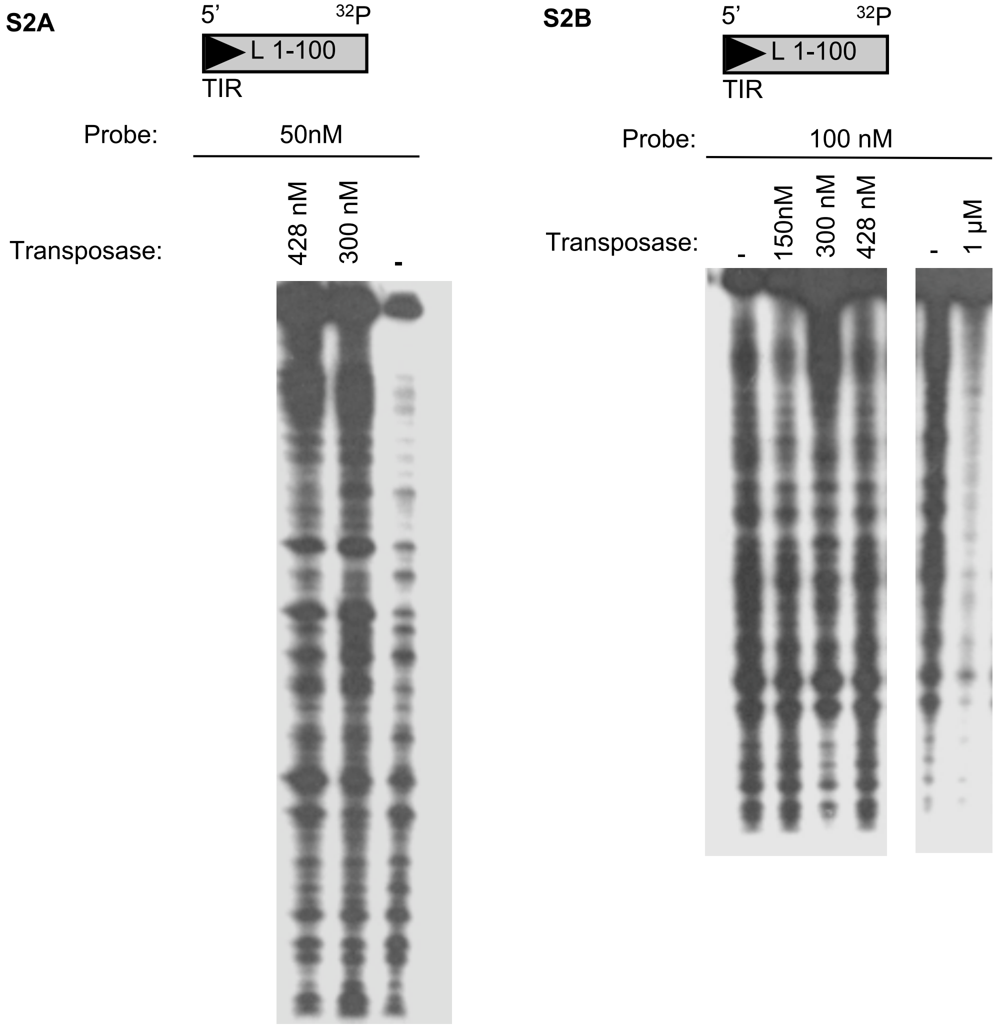

Figure S2.

A) 50 nm or **B)** 100nM of the single-end labeled *Herves*-L 1-100bp fragment was incubated in absence (-) or presence of the transposase at various concentrations as indicated. ^{32}P indicates end of the probe that was labeled.
