# Supplementary material for: DNA binding activities of the Herves transposase from the mosquito Anopheles gambiae
Source: Mob DNA. 2011 Jun 20;2:9. doi: 10.1186/1759-8753-2-9 (PMC3143072; doi:10.1186/1759-8753-2-9)
Supplement: Additional file 3 — Figure 3with DNA ladder. The panel is identical to that shown in the left of Figure 3 but with a DNA ladder. The nucleotide positions were determined by the Sanger sequencing reactions shown in lanes G and A. [file 1759-8753-2-9-S3.PDF]

Additional File 3, Figure S3

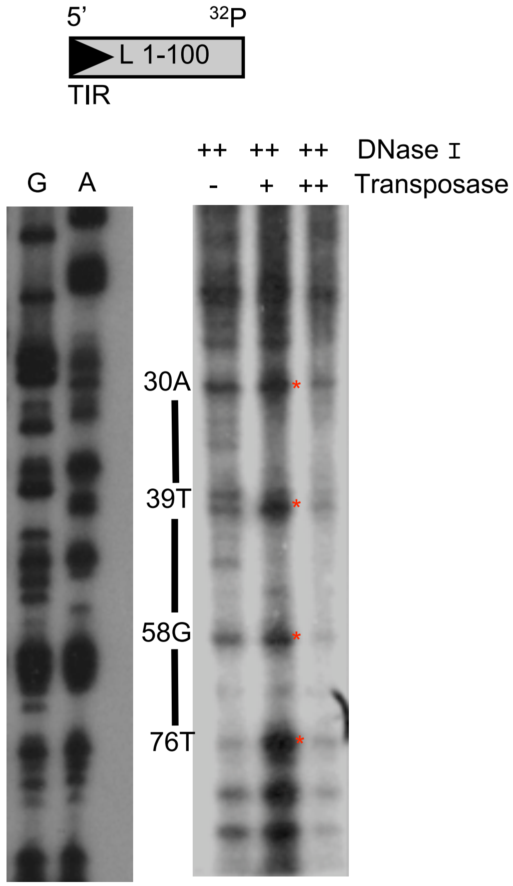

Figure S3.

The panel is identical to that shown in the left of Figure 3 but with a DNA ladder. The nucleotide positions were determined by the Sanger sequencing reactions shown in lanes G and A.
